# The Tibetan Plateau Uplift is Crucial for Eastward Propagation of Madden-Julian Oscillation

**DOI:** 10.1038/s41598-019-51461-w

**Published:** 2019-10-29

**Authors:** Young-Min Yang, June-Yi Lee, Bin Wang

**Affiliations:** 1 Key Laboratory of Meteorological Disaster of Ministry of Education and Earth System Modeling Center, Nanjing University of Information Science and Technology, Nanjing, China; 2 Department of Atmospheric Sciences and International Pacific Research Center, University of Hawaii, Honolulu, Hawaii 96822 USA; 3 Research Center for Climate Sciences and Department of Climate System, Pusan National University, Busan, 46241 Republic of Korea; 4 Center for Climate Physics, Institute for Basic Science (IBS), Busan, 46241 Republic of Korea

**Keywords:** Climate and Earth system modelling, Atmospheric dynamics

## Abstract

The Tibetan Plateau (TP) and Himalayas have been treated as an essential external factor in shaping Asian monsoon and mid-latitude atmospheric circulation. In this study we perform numerical experiments with different uplift altitudes using the Nanjing University of Information Science and Technology Earth System Model to examine potential impacts of uplift of the TP and Himalayas on eastward propagation of the MJO and the associated mechanisms. Analysis of experimental results with dynamics-based MJO diagnostics indicates two potential mechanisms. First, the uplift considerably enhances low-level mean westerlies in the Indian Ocean and convection in the Maritime Continent, which in turn strengthens boundary layer moisture convergence (BLMC) to the east of the MJO convective center. The increased BLMC reinforces upward transport of moisture and heat from BL to free atmosphere and increases lower tropospheric diabatic heating by shallow and congestus clouds ahead of the MJO center, enhancing the Kelvin-Rossby wave feedback. Second, the uplift increases upper tropospheric mean easterlies and stratiform heating at the west of the MJO center, which contributes to eastward propagation of MJO by generating positive moist static energy at the east of MJO center. This study will contribute to a better understanding of the origin of the MJO and improvement in simulation of MJO propagation.

## Introduction

The Madden Julian oscillation (MJO)^1^, the eastward propagating convection-circulation coupled system along the equator with 20–70-day time scales, has been received prodigious attention for the last few decades due to its extensive impact on extreme weather and climate events worldwide^2–5^. A better understanding of the generation, propagation and impact of the MJO is one of the big challenges in climate sciences. Among the many theories proposed to explain MJO propagation, the trio-interaction theory between diabatic heating, moisture and equatorial waves dynamics^6^ paved the way to provide the integrated view on the MJO propagation mechanism. By linking the coupled Rossby-Kelvin wave dynamics^7^ with moisture mode theory^8^, the trio-interaction theory suggests that the primary driver for the MJO eastward propagation is the eddy available potential energy (EAPE) generation to the east of the MJO convective center, which is induced by the atmospheric boundary layer (BL) dynamics and its interaction with lower tropospheric heating as well as coupled Rossby-Kelvin wave dynamics^6,9^.

The Tibetan plateau (TP) and Himalayas play critical roles in shaping the Asian-Australian monsoon system^10–12^ and middle-latitude atmospheric circulation in the Northern Hemisphere (NH)^13–16^ since their tectonic uplift during the middle and late Cenozoic from about 20 to 50 million years ago^14,17^. Numerical experiments with varying elevations of the TP and Himalayas in previous studies suggest that the tectonic uplift disturbed the zonally oriented atmospheric flow, enhanced the continentality of climate such as cold winters, and caused greater east-west differentiation and more uneven seasonal precipitation^13–15,18^. In particular, many modeling studies strongly supported the idea that the thermal and mechanical effects of the TP and Himalayas are the most crucial factor in the formation, evolution, amplitude and variability of the Asian summer monsoon system^18–21^.

Previous studies focused on the changes in mean-state circulation and precipitation over the Asia region driven by the mountain uplift. However, how the uplift modulates amplitude and propagation of intraseasonal oscillation has not been well discussed. Given the numerical evidence that the TP and Himalayas should significantly modulate mean sea surface temperature (SST) and atmospheric BL moisture distribution^18^ in addition to the mean atmospheric circulation and energetics^15^, a question arises as to whether the uplift also influences the intraseasonal oscillation such as the MJO. To answer that, this study explores, for the first time, the role of the tectonic uplift on the characteristics of MJO during boreal winter by performing numerical experiments with varying elevation of the uplift using the Nanjing University of Information Science and Technology Earth System Model^22^ (NESM3.0). Experiments include: control simulation using observed elevation (TP100) (Fig. S1a); simulation with reduced orography by 50% (TP50) (Fig. S1b); and simulation with no uplift over the TP and Himalayas (TP0). The dynamics-based MJO diagnostics^23,24^ are applied to the analysis of numerical experimental results (See ‘Methods’ for details of observed data, model, experiment design, and the dynamics-based MJO diagnostics.).

## The TP Modulation On The MJO Propagation

**Figure 1:**
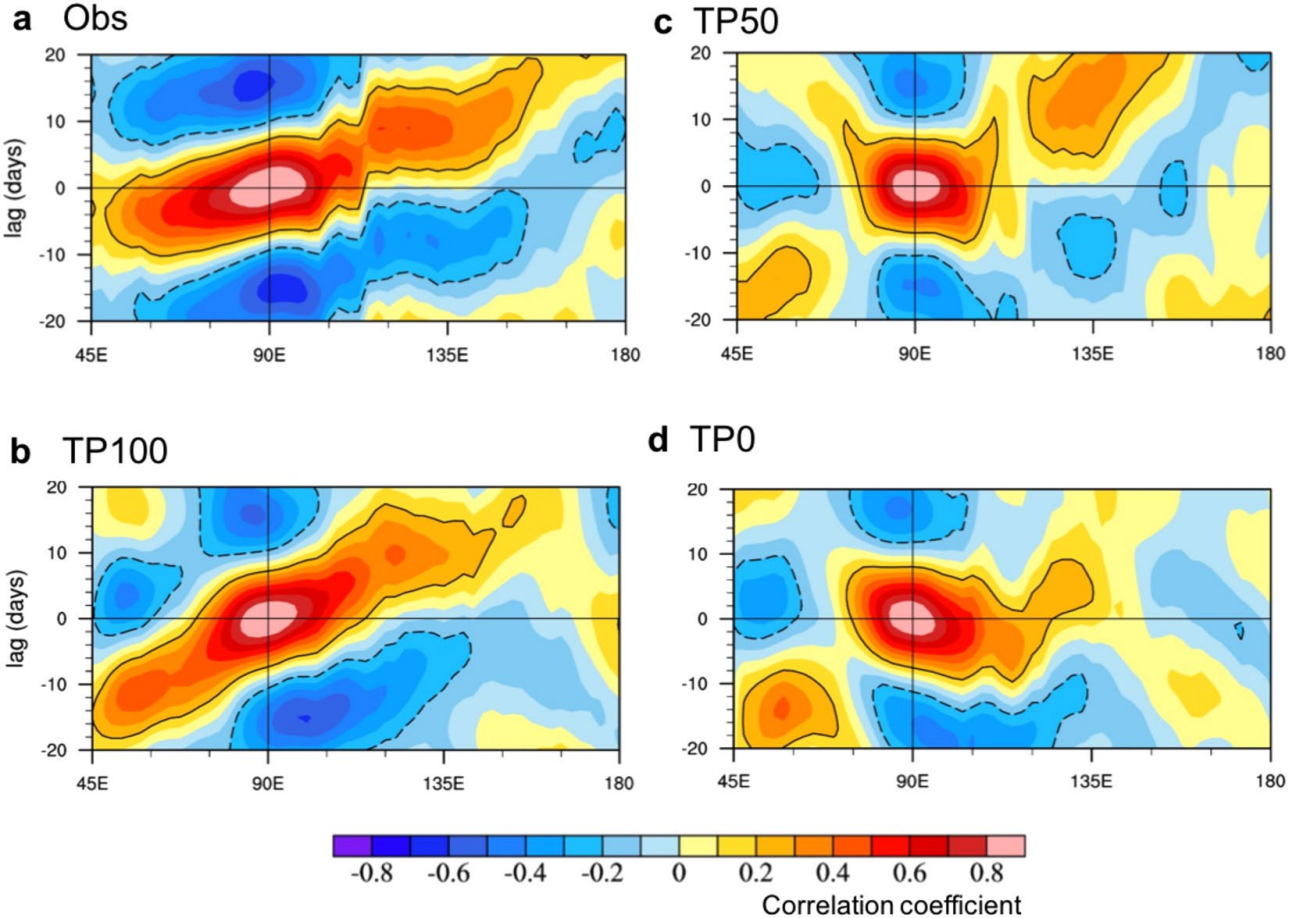
The characteristics of Madden-Julian Oscillation (MJO) propagation in observation and model simulations. The lead-lag correlation of 20–70-day filtered precipitation averaged over 10°S–10°N with reference to the filtered precipitation at the MJO convective center over the equatorial Indian Ocean (10°S–10°N, 80°–100°E) during boreal winter from November to April (NDJFMA) derived from (**a**) observation (OBS) and model simulations with (**b**) the observed height (TP100), (**c**) the 50%-reduced height (TP50), and (**d**) no uplift (TP0). The black contour represents the correlation coefficient at 95% confidence level.

**Figure 2:**
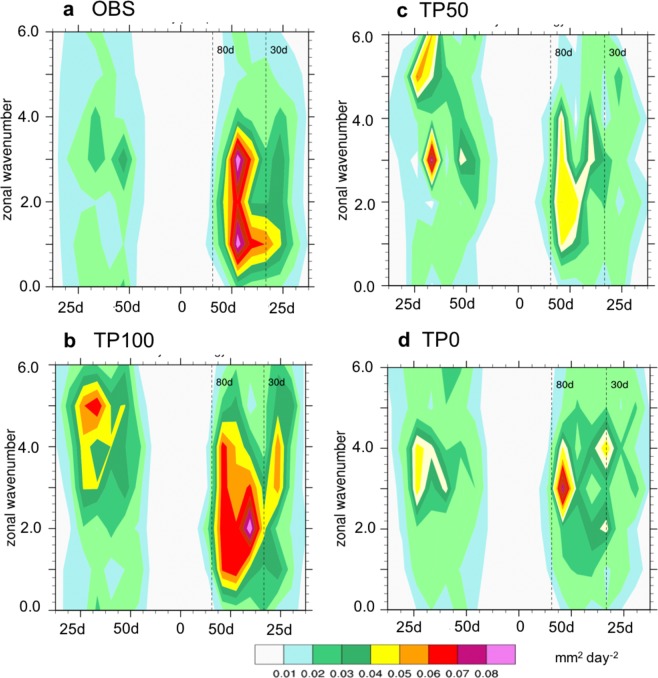
Wavenumber-frequency spectra of precipitation averaged over 10^o^S–10^o^N. The spectra of 20–70-day filtered daily precipitation (unit: mm^2^ day^−2^) obtained from (**a**) observation and model simulations with (**b**) TP100, (**c**) TP50, and (**d**) TP0 during NDJFMA. The climatological seasonal cycle and time mean were removed before the calculation of the spectra.

Analysis of the numerical experiments performed in this study suggests that the uplift of the TP and Himalayas exerts a considerable influence on the MJO. On the one hand, robust intraseasonal variability during boreal winter exists regardless of the uplift of the TP and Himalayas, however, the uplift alters the amplitude of intraseasonal variability depending on region (Fig. S2). In the TP50 and TP0, the variance of the 20–70-day filtered daily precipitation from November to April decreased slightly in the Indian Ocean and the western North Pacific but increased in the western South Pacific compared with that of the TP100. On the other hand, note that the eastward propagating MJO can be generated only with the existence of the TP and Himalayas, while non-propagating or westward propagating MJO is dominant without the uplift (Figs 1 and 2). Figure 1 displays the lead-lag correlation of 20–70-day filtered daily precipitation averaged over 10°S and 10°N with reference to the filtered precipitation at the MJO convective center over the equatorial Indian Ocean (10°S–10°N, 80°–100°E). In the observation, the MJO propagates eastward at a speed of about 5 m s^−1^ for both wet and dry events. With the realistic TP uplift (TP100), the model simulation clearly shows eastward propagation at a slower speed compared to observation (Fig. 1b). However, the eastward propagation is much weakened in the TP50 (Fig. 1c) and TP0, and slight westward propagation is shown over the Maritime Continent (from 90° to 120°E) (Fig. 1d). Note that the propagation characteristics of the MJO in the three experiments and observation are not sensitive to latitudinal location of the reference MJO precipitation (see Fig. S3).

Figure 2, which depicts wavenumber-frequency spectra of 20–70-day filtered daily precipitation along the equator (10°S–10°N), further demonstrates that the TP uplift intensifies intraseasonal variability mainly with eastward propagation. Observation shows a strong eastward propagation at the 30–70-day period and zonal wave number 1–3. Compared to eastward propagation, westward propagation is much weaker and its horizontal scale is smaller (zonal wave number 3–4). In the TP100, eastward propagation is much stronger than westward propagation at 30–70-day period and zonal wave number 1–3, implying that the TP100 realistically captures the eastward propagating MJO with planetary horizontal scale similar to observation. However, the TP50 simulates a reduced eastward-propagating power at the 50–70-day period and zonal wave number 1–3. It also shows a relatively strong westward propagation at the 20–30-day period and zonal wave number 3 and 5, suggesting that the signal of the MJO eastward propagation is weakened and westward propagation with small horizontal scale intensified. Note that this feature is consistent with those shown in Fig. 1c. The TP0 shows the peak of the 60–70-day period and zonal wave number 3 for eastward propagation, suggesting that eastward propagation speed is much slower and its horizontal scale is smaller than that of TP100. There is a westward propagation during the 40-day periods and zonal wave number 3 and 4. These results indicate that lowering TP may generate both weak eastward and westward propagation with smaller horizontal scale, and thus the MJO propagation signal from the lead-lag correlation shown in Fig. 1c,d is highly suppressed.

## Mechanisms Controlling The Changes In MJO Propagation

**Figure 3:**
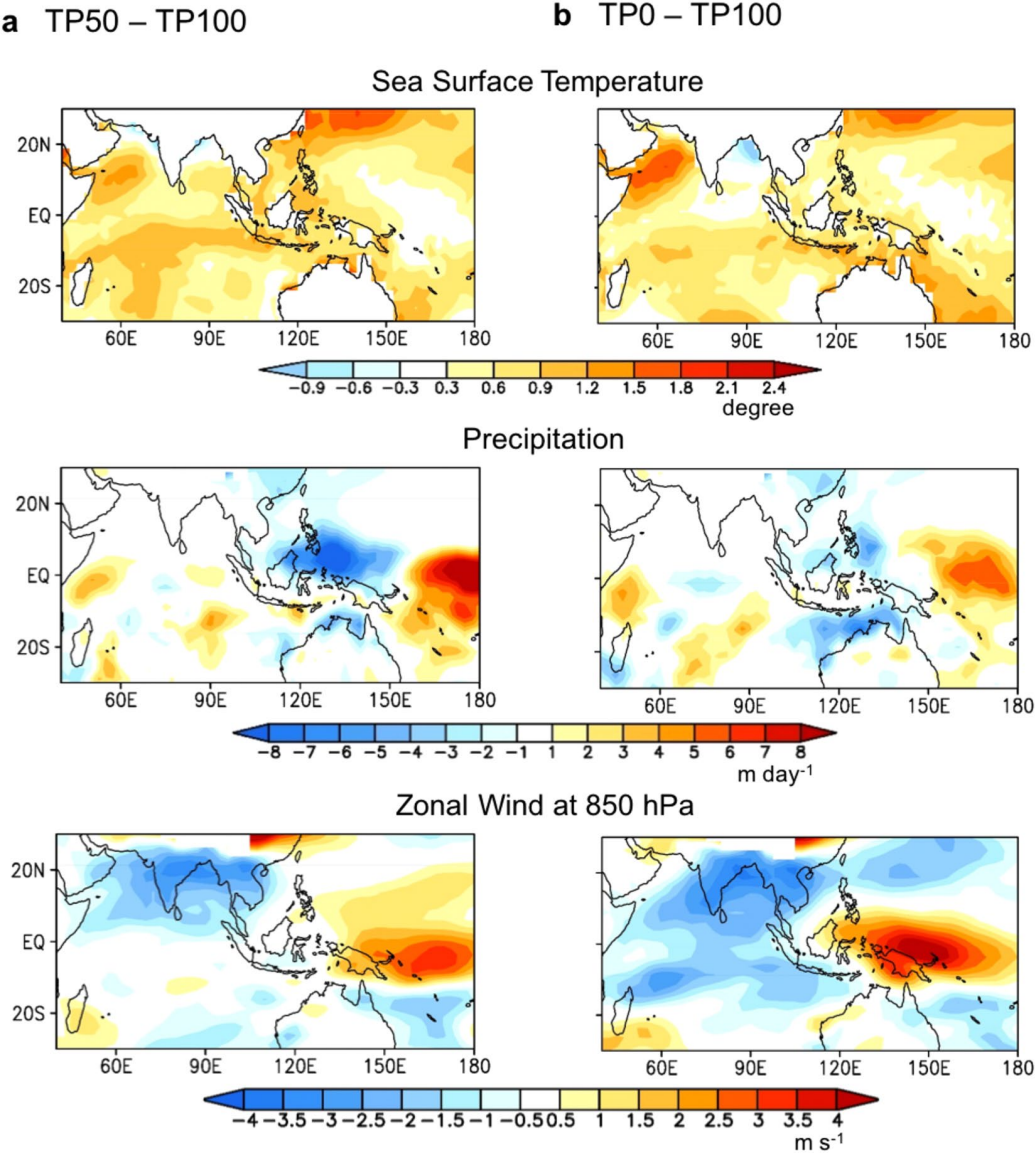
Changes in mean states due to the TP uplift. Difference of DJF sea surface temperature (unit: degree, upper panels), precipitation (unit: mm day^−1^, middle panels), and 850-hPa zonal wind (unit: m s^−1^, lower panels) between (**a**) TP50 and TP100 and (**b**) TP0 and TP100.

Here we show that the TP uplift provides more favorable conditions in promoting the eastward propagation of MJO via changing mean state of SST, precipitation, and circulation. Figure 3 shows that lowering TP elevation increases SST over the Indian Ocean, decreases precipitation over the Maritime Continent and western Pacific, and forms anomalous low-level easterlies in the Indian Ocean and westerlies in the western Pacific. In the TP50, the SST is 1.0 K warmer than that of TP100 in the western and equatorial Indian Ocean but there is no significant difference in the western Pacific (Fig. 3a, upper panel). The mean change in SST reduces the zonal SST gradient between the western and eastern Indian Ocean, which may cause a decrease in the MJO amplitude in the Indian Ocean (Fig. S2e,f). Precipitation significantly decreases over the Maritime Continent and western Pacific due to the change in SST (Fig. 3a, middle panel), which induces a sinking motion over the western Pacific and then anomalous surface easterlies^22^ over the Indian Ocean (Fig. 3a, lower panel). In the TP0, the horizontal patterns of SST difference are similar to those in the TP50 except in the Arabian Sea (1.5 K). There are significant anomalous easterlies in the Indian Ocean and westerlies in the western Pacific (Fig. 3b, lower panels). The anomalous sinking motion and low-level divergence over the Maritime Continent and western Pacific due to the lowering TP are unfavorable conditions for the MJO eastward propagation from the Indian Ocean into Pacific.

**Figure 4:**
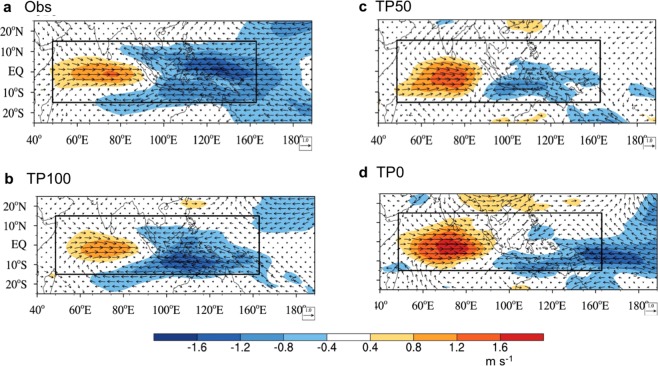
The MJO circulation structure at 850 hPa. Horizonal structure of horizontal wind (unit: m s^−1^, vector) and zonal wind speed (unit: m s^−1^, shading) at 850 hPa depicted by the regressed 20–70-day filtered wind onto the 20–70-day filtered precipitation averaged over the MJO precipitation center (10°S–10°N, 80°–100°E) obtained from (**a**) observation and model simulations with (**b**) TP100, (**c**) TP50, and (**d**) TP0 during boreal winter (November to April). The regression strengths are scaled to a fixed 3 mm day^−1^ precipitation rate.

We further investigate linkages between the mean-state changes due to the uplift and changes in the MJO characteristics by applying the dynamics-based MJO diagnostics^24^ including: MJO circulation structure; boundary layer moisture convergence (BLMC); equivalent potential temperature (EPT); diabatic heating; and EAPE. We first examine changes in MJO circulation structure as a function of the TP elevation (Fig. 4). Observation shows a zonally symmetric wind about the equator and a coupled Rossby-Kelvin wave structure in the Indian Ocean. The ratio of equatorial maximum westerly speed to the west of the MJO center to easterly speed to the east of the MJO center is 1.2. The TP100 simulation captures the observed circulation patterns. The ratio of maximum westerly to easterly wind is similar to observation. In the TP50, the westerly wind associated with MJO is strengthened but easterly wind is slightly reduced. The ratio is larger than the TP100 and observation. In the TP0, the horizontal circulation pattern differs from the observed MJO: zonal wind is not symmetric; westerly is much stronger than the easterly; and the ratio is larger. The lowering TP reduces the mean surface westerly in the Indian Ocean as shown in Fig. 3 (lower panels). The reduced westerly mean wind weakens generation of a coupled Rossby-Kelvin wave and reduces the low-pressure anomaly to the east of the MJO convection center, thus decreasing the BLMC and lower tropospheric moistening to the east of the MJO convection. This decreases the diabatic heating in the lower troposphere to the east of the MJO major convective center and could further weaken MJO-associated circulation such as a coupled Kelvin-Rossby wave system.

The Rossby-Kelvin wave feedback can generate the low in the northwest, southwest and the east of the MJO convective center. The weakened wave feedback and anomalous easterly wind in the TP50 and TP0 could reduce the BLMC to the east of the MJO convective center. Figure S4 displays the lead-lag correlation of 20–70-day filtered BLMC averaged over 10°S and 10°N with reference to the filtered precipitation at the MJO convective center over the Indian Ocean (10°S–10°N, 80°–100°E). In observation, the BLMC propagates eastward from the western Indian Ocean to the dateline. The eastward propagation of BLMC is also highly correlated with those of the MJO precipitation with 5-day lead-time. This suggests that BLMC drives the eastward propagation of the MJO deep convection. The TP100 reproduces the observed eastward propagation of the BLMC well but the TP50 and TP0 fail to capture the eastward propagation.

The BLMC induces vertical motion, which transports moisture and heat in the boundary layer to the lower troposphere^25,26^. We further analyze EPT, which represents moist static energy and the moist thermodynamic structure of the MJO^9^ and can indicate moistening processes and convective instability. Figure S5 displays the vertical profiles of the EPT from observation and three model simulations. Observation shows the eastward extension of the EPT in the lower troposphere, signifying that the pre-moistening occurs ahead of the next major MJO convection at the lower troposphere. The TP100 captures the eastward and downward tilt structure of the EPT in the lower troposphere reasonably well. The TP50, however, fails to reproduce the eastward and downward tilt structure and has much weaker magnitude of the EPT than observation. In the TP0, moistening associated with the convection occurs more broadly in depth to the east of the MJO center without the tilt structure shown in observation and the TP100.

An abundance of moisture in the lower troposphere at the east of MJO convection center can induce shallow or congestus cloud, which generates lower tropospheric heating and further moistens by enhancing a vertical motion^27^. Figure S6 shows the vertical structure of diabatic heating associated with the MJO convective center. The observation shows an eastward extension of diabatic heating in the lower troposphere, signifying the occurrence of shallow and congestus clouds before development of the MJO deep convection and their gradual change to deep convection. The TP100 realistically simulates the extension of diabatic heating with relatively stronger magnitude. There is a strong easterly (westerly) wind in the east (west) of the MJO convection in the lower atmosphere. In the TP50, the extension of lower tropospheric diabatic heating is weaker than in the TP100. Compared to the TP100, the corresponding easterly (westerly) to the east (west) of the MJO convection is weaker and downward motion in the western Pacific is stronger in the TP50. The TP0 experiment does not simulate the lower tropospheric diabatic heating at the east of the MJO convection and fails to generate the shallow and congestus clouds before MJO deep convection. Our results suggest that the TP50 and TP0 are limited in capturing the gradual transition from shallow and congestus clouds to the major deep convection^28,29^.

**Figure 5:**
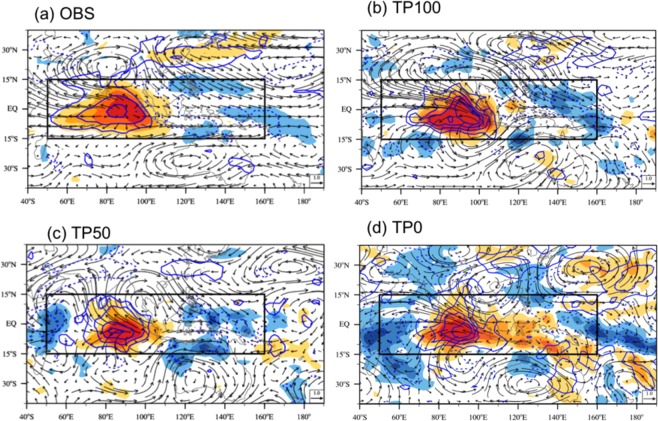
Diabatic heating (300 hPa, shading, K day^−1^), divergence (200 hPa, contour, day^−1^) and wind (200 hPa, vector, m s^−1^) from the observations and model experiments. The horizontal structures show regressed 20–70-day filtered 200-hPa divergence and wind, and 300-hPa diabatic heating onto the 20–70-day filtered precipitation at the equatorial EIO (10°S–10°N, 80°–100°E). The regressed strength is scaled to a fixed 3 mm day^−1^, The black box line shows the area used for pattern correlation score.

Lifting the TP can also change upper tropospheric circulation, which can affect MJO eastward propagation^9,24,30,31^. Figure 5 shows 300-hPa diabatic heating (shading) and 200-hPa winds (vector) and divergence (contour) associated with the MJO convection center (90°E). In observation, there is a relatively strong anti-cyclonic Rossby wave component (and associated equatorial easterly anomalies) and relatively weak Kelvin wave component (and equatorial westerly anomalies). The upper-level circulation pattern is almost out of phase with the lower tropospheric circulation. The observation also shows that the 200-hPa divergence pattern resembles that of the 300-hPa diabatic heating. The peaks of diabatic heating and divergence are located at the MJO convective center but extend westward from the MJO center. The westward extension of upper-level heating represents existence of stratiform heating, which induces negative MSE tendency to the west of MJO center, favoring MJO eastward propagation. The TP 100 reproduces the observed pattern of 200-hPa divergence and associated circulation, including the westward extension of 300-hPa diabatic heating from MJO deep convection. However, the TP50 fails to capture the westward extension of diabatic heating and divergence; rather it shows diabatic cooling and convergence at the west of the MJO center. The Kelvin wave westerly anomalies are weakened and anticyclonic anomalies to the west of MJO center are not observed. The TP0 simulates a stronger eastward extension of the upper tropospheric diabatic heating to the east of the MJO center and a more westward extension of the diabatic cooling to the west of the MJO center than those of the TP50. The Kelvin wave westerlies are not seen at east of the MJO center. The horizontal pattern of diabatic heating and divergence is far from observation and may be unfavorable for MJO propagation. It is interesting to note that the mean change in the upper-level circulation, e.g., anomalous westerly, to the west of the MJO center in the TP50 and TP0 resembles the westerly phase of the quasi-biennial oscillation (QBO), which is not favorable for the amplitude and eastward propagation of the MJO compared to the QBO easterly phase^31,32^. One of key factors for the amplification and better propagation of the MJO during the QBO easterly phase is the stronger cold tropical tropopause layer superimposed to the MJO convective center than its westerly phase^31^. Figure S5 clearly shows that the cold tropical tropopause layer is much stronger in observation and TP100 than the TP50 and TP0, indicating the upper-level circulation change due to the TP uplift indeed plays a critical role on amplitude and eastward propagation of the MJO.

## Summary and Discussion

By changing the level of uplift in the fully coupled experiments, this study investigates the influence of the TP and Himalayas on MJO characteristics, and tries to better understand how the topography affects the MJO eastward propagation. The model experiments show that the realistic orography over the TP and Himalayas drastically improves the MJO eastward propagation compared to those with flat topography. It also affects not only MJO basic characteristics (e.g., wave-power spectra) but also three-dimensional dynamic and thermodynamic structures of the MJO attributable to the changes in mean state of SST, precipitation, and tropospheric circulation. The major amplification of the MJO with realistic topography occurs at the 20–70-day timescales with wavenumber 1–3.

This study finds two pathways to the enhancement of the MJO eastward propagation from the mean state changes in SST, precipitation, and tropospheric circulation due to the uplift of TP and Himalayas. On the one hand, the uplift modulates the dynamic and thermodynamic structure and eastward propagation of the MJO by changing the mean state of low-level zonal wind, SST, and precipitation. On the other hand, the uplift affects the MJO propagation by changing the upper-tropospheric mean circulation, temperature and stratiform heating.

The former is mainly associated with low-tropospheric mean changes in the Indian Ocean and Maritime Continents, and can be understood by utilizing the trio-interaction theory^6,7,9,24^. This theory considers the interaction among convective heating, moisture and wave-BL dynamics, which is essential for the MJO dynamics. Lowering TP reduces the mean surface westerly in the Indian Ocean, which weakens generation of a coupled Rossby-Kelvin wave and also the low-pressure anomaly to the east of the MJO convection center, thus decreasing the BLMC to the east of the MJO convection. The weakened BLMC reduces upward transport of moisture from the BL to lower troposphere, and therefore decreases the diabatic heating in the lower troposphere to the east of the MJO major convection. The reduced lower troposphere heating represents suppression of the shallow and congested clouds. Observations show that increased moistening in the lower troposphere (pre-moistening), increased convective instability (pre-destabilization), and increased shallow-congestus clouds lead to MJO deep convection (multi-cloud structure) and provide favorable conditions for MJO eastward propagation^33–40^. Therefore, the reduced TP topography weakens eastward propagating MJO. In addition, the decreased mean precipitation in the Maritime Continents and associated low-level divergence may prevent from the MJO propagation from the Indian Ocean to western Pacific.

The latter is related to upper-tropospheric mean changes to the west and middle of the MJO convective center. In observation, the stratiform heating occurring west of the MJO convection can induce negative MSE tendency to the west of the MJO deep convection, while the lower tropospheric shallow convective heating to the east of the MJO center by BLMC can generate positive MSE^30^. This eastward tilted vertical heating structure in the lower troposphere is favorable for development of subsequent deep convection to the east of the MJO center. The lowering TP tends to weaken the heating to the west of MJO center through reducing the upper-level easterly mean wind. The weakened stratiform heating may result from the weakened transport of moisture to the west of the MJO center, which is unfavorable for MJO eastward propagation. The upper-tropospheric circulation change in the TP50 and TP0 resembles the westerly phase of QBO, which is not favorable for the MJO amplification and propagation compared to its easterly phase. In particular, the anomalous cold tropical tropopause layer, which is key for MJO amplification and propagation in the QBO easterly phase, is much stronger in the observation and TP100 than in TP50 and TP0.

We further examine the possibility that the uplift of the TP and Himalayas possibly increases the energy source of the MJO disturbance by analyzing the EAPE (Fig. S7). It has been suggested that the EAPE generation contributes to the amplification and maintenance of the MJO strength^41^. We suggest that the total amount of the EAPE generation integrated within the MJO system may determine the amplification or decay of the system at a given time, but its spatial distribution, especially the east-west asymmetry in EAPE generation should be related to the propagation at that given time. We speculate that the generation of EAPE to the east of MJO and dissipation of EAPE to the west of the MJO likely change MJO eastward propagation because the generated EAPE to the east of the MJO center can be converted to kinetic energy by enhanced upward motion, which induces subsequent development of convection to the east of the MJO deep convection (e.g., Fig. S7a,b). Because the EAPE is defined as covariance of temperature and diabatic heating anomalies^22^ and the vertical structure of EAPE is similar to that of the diabatic heating profile, a change in the diabatic heating (or BLMC and moistening) profile may be a possible reason for the change of MJO eastward propagation.

This study will contribute to a better understanding of MJO origin and propagation and also a better simulation of MJO propagation. It will be worth conducting experiments using different climate models to examine model dependency because the MJO simulation is sensitive to convective parameterizations as well as other physical processes (e.g., radiative processes, BL diffusion and stratiform cloud scheme).

## Methods

### Earth system model (ESM)

We use the third version of the Nanjing University of Information Science and Technology (NUIST) Earth System Model (NESM3.0). The NESM3.0 includes atmosphere, ocean, land and sea ice components that are fully coupled with each other. The resolution of the atmosphere and land model is T63L47. The horizontal resolution of ocean model is 1° grid and is refined to 1/3° over the equatorial region. The vertical resolution of the ocean model is 46 vertical layers and it has 15 layers from the surface to 100 meters depth. The NESM3.0 simulates not only reasonable climatology but also key characteristics of MJO^22^, although the eastward propagation signals are slightly overestimated.

### Design of numerical experiments

Three experiments were conducted with varying elevations of the TP and Himalayas using the NESM3.0 to examine the impact of tectonic uplift on the MJO as follows: (1) control experiment with the observed orography (TP100) (Fig. S1a); (2) experiment with orography reduced by 50% (TP50) (Fig. S1b); and (3) experiment with no uplift over the TP and Himalayas (TP0). Note that the orography at the boundaries is smoothed to inhibit numerical noise. There is no other tuning of parameterization in the simulations. For all experiments, 50-year simulations were conducted using fixed 1990’s external forcings, which includes greenhouse gases, solar constant, aerosol concentration, and ozone. We used the last 20 years of data for analysis.

### The observed data and diagnostic methods

Observed circulation data are obtained from the European Center for Medium-Range Weather Forecast Reanalysis (ERA) Interim data^42^. The Global Precipitation Climatology Project (GPCP) daily data^43^ are used for the precipitation data (1997–2014). We applied a 20–70-day band-pass filter to obtain the MJO signal. We focused on the boreal winter season (November 1st to April 30th) because of significant seasonal dependency in tropical intraseasonal variability^44^.

Two MJO diagnostic methods are used in the study. The first is the basic MJO diagnostic suggested by Climate Variability and Predictability Program (CLIVAR) MJO Working Group (MJO WG)^23^, which includes: (1) climatological precipitation, SST and surface winds; (2) total (or filtered) variance; and (3) space-time spectrum of precipitation. The second is dynamic and thermodynamic oriented diagnostics^24^, which include: (1) horizontal structure of boundary layer moisture convergence; (2) horizontal structure of 850-hPa zonal wind and its equatorial asymmetry; (3) vertical structure of equivalent potential temperature; (4) horizontal and vertical structure of diabatic heating; and (5) generation of the MJO eddy available potential energy.

## Supplementary information

Supplimentary Figures

## Data Availability

All observed data and model experiment data are available upon request. All observed data used in this study are publicly available and new data generated in this study are available upon request.
