# Supplementary material for: The Tibetan Plateau Uplift is Crucial for Eastward Propagation of Madden-Julian Oscillation
Source: Sci Rep. 2019 Oct 29;9:15478. doi: 10.1038/s41598-019-51461-w (PMC6820537; doi:10.1038/s41598-019-51461-w)
Supplement: Supplementary file 1 — Supplimentary Figures [file 41598_2019_51461_MOESM1_ESM.docx]

**Supplementary Information**

**The Tibetan Plateau Uplift is Crucial for Eastward Propagation of Madden-Julian Oscillation**

Young-Min Yang^1,2^, June-Yi Lee^*3,4^ and Bin Wang ^1,2^

^1^ Key Laboratory of Meteorological Disaster of Ministry of Education and Earth System Modeling Center, Nanjing University of Information Science and Technology, Nanjing, China

^2^ Department of Atmospheric Sciences and International Pacific Research Center, University of Hawaii, Honolulu, Hawaii 96822, USA

^3^ Research Center for Climate Sciences and Department of Climate System, Pusan National University, Busan, Korea, 46241

^4^ Center for Climate Physics, Institute for Basic Science (IBS), Busan, South Korea, 46241

The supplementary information contains supplementary figures S1-S7.


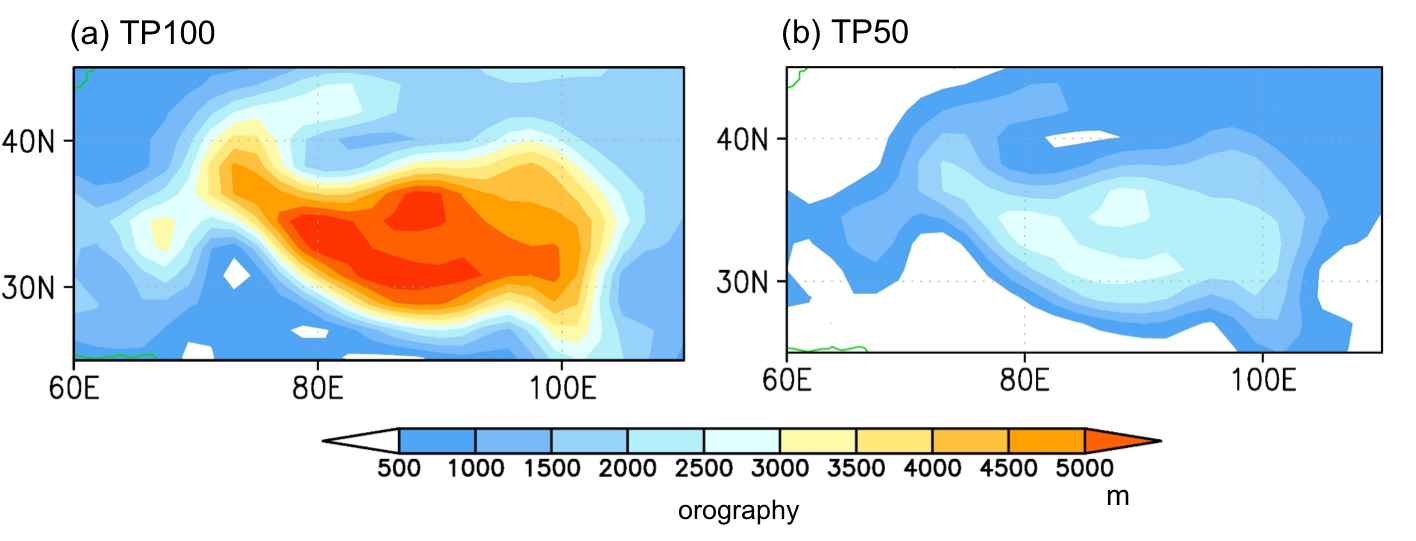


**Figure S1.** Horizontal map of orography (unit: m) over the Tibetan Plateau (TP) and Himalayas used in the model experiments with (a) the observed (TP100) and (b) the 50%-reduced height (TP50).


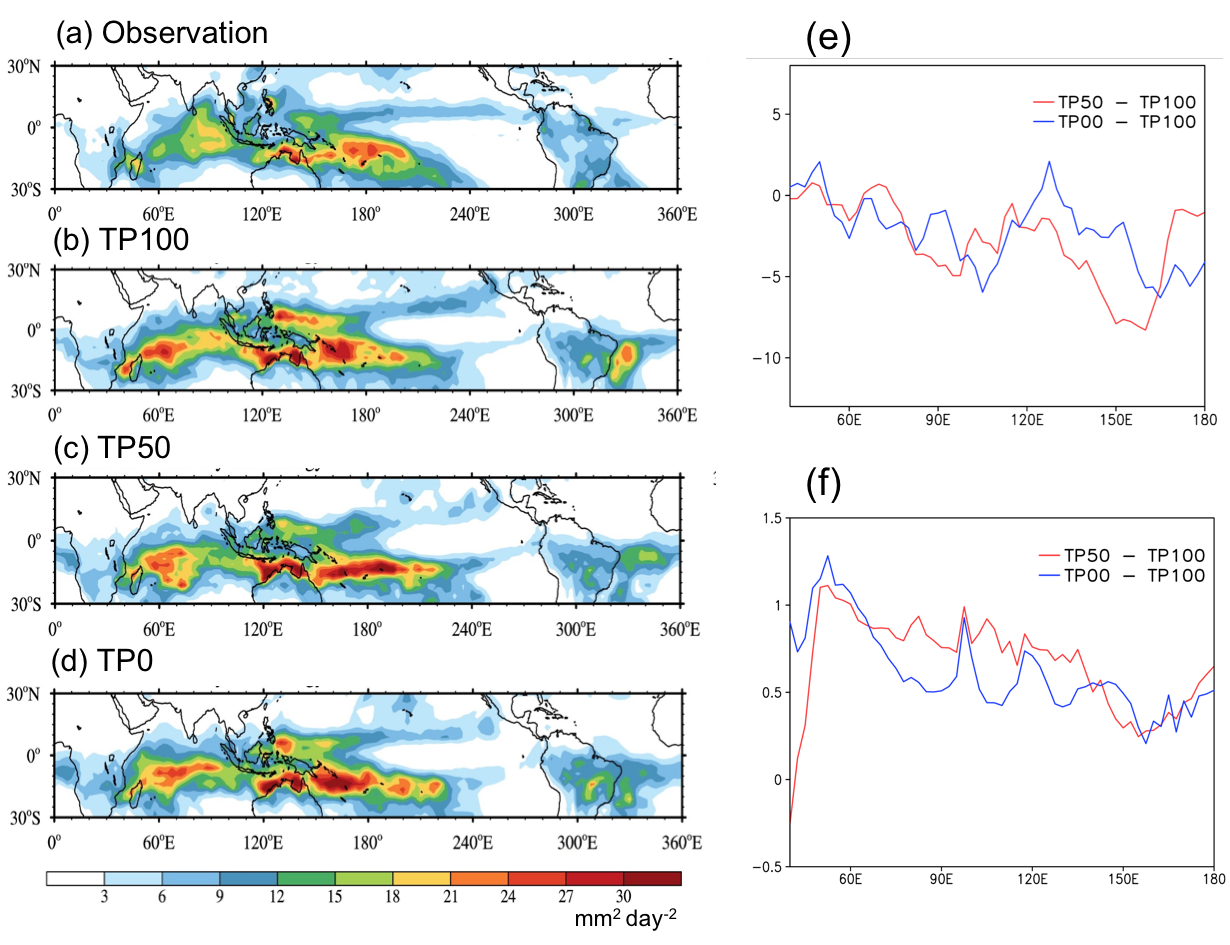


**Figure S2.** Variance of 20-70-day filtered daily precipitation from November 1^st^ to April 30^th^ obtained from (a) observation and three experiments of (b) TP100, (c) TP50 and (d) TP0. The unit is mm^2^ day^-2^. e) difference of MJO varaince and f) SST averaged over 10^o^S and 10^o^N between TP0 (or TP50) and TP100


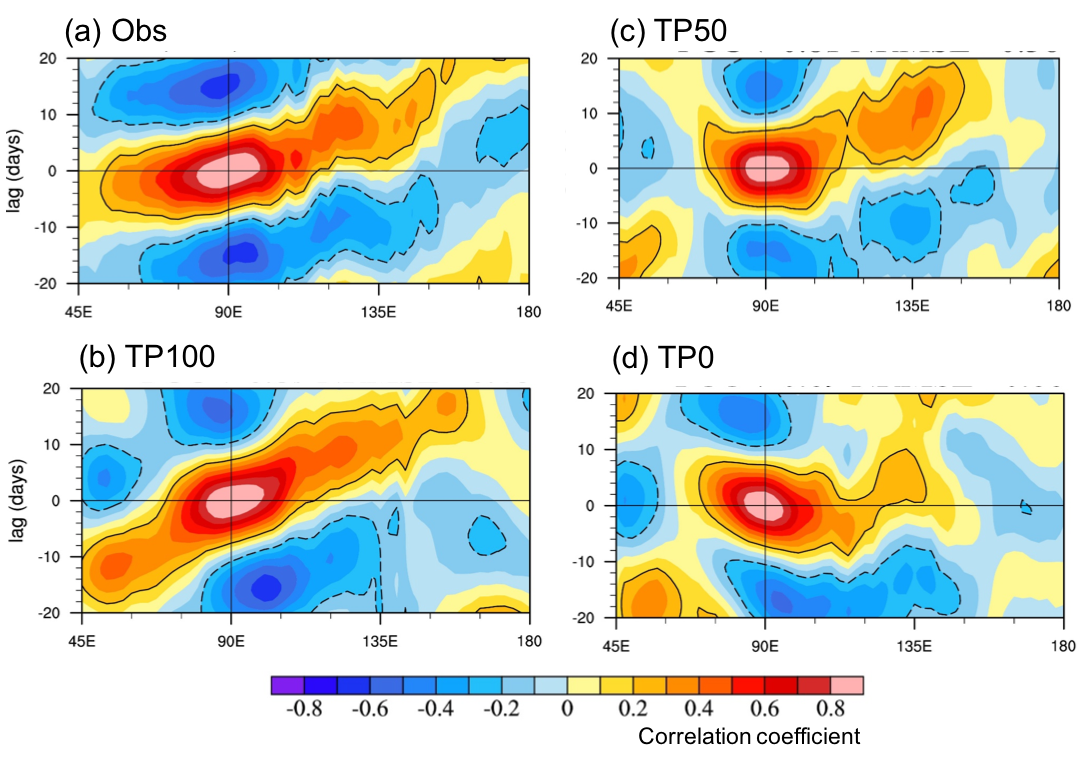


**Figure S3.** Same as Figure 2 except for the reference point averaged over 10^o^S-eq, 80^o^-100^o^E.


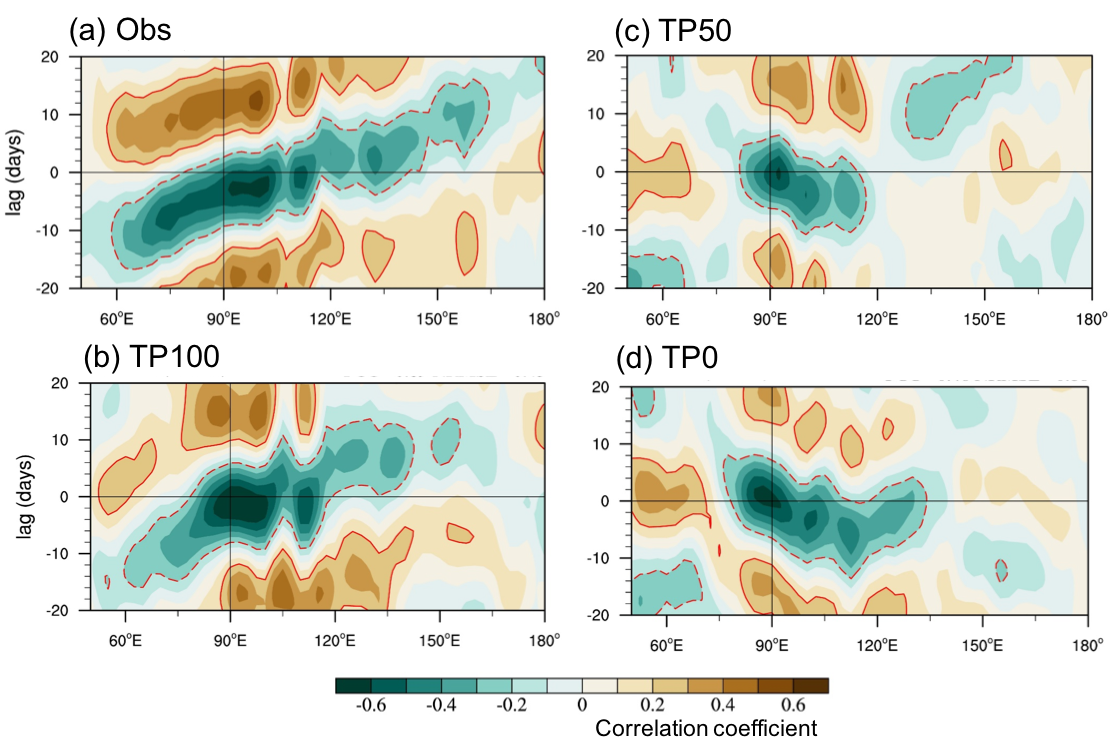


**Figure S4.** Propagation of BLMC (925 hPa) as depicted by the lead-lag correlation of 20-70 day filtered BLCM averaged over 80°-100°E with reference to the precipitation at the MJO convective center over the equatorial Indian Ocean (10°S-10°N, 80°-100°E) during NDJFMA derived from (a) observation and model simulations in the experiments with (b) TP100, (c) TP50 and (d) TP0.


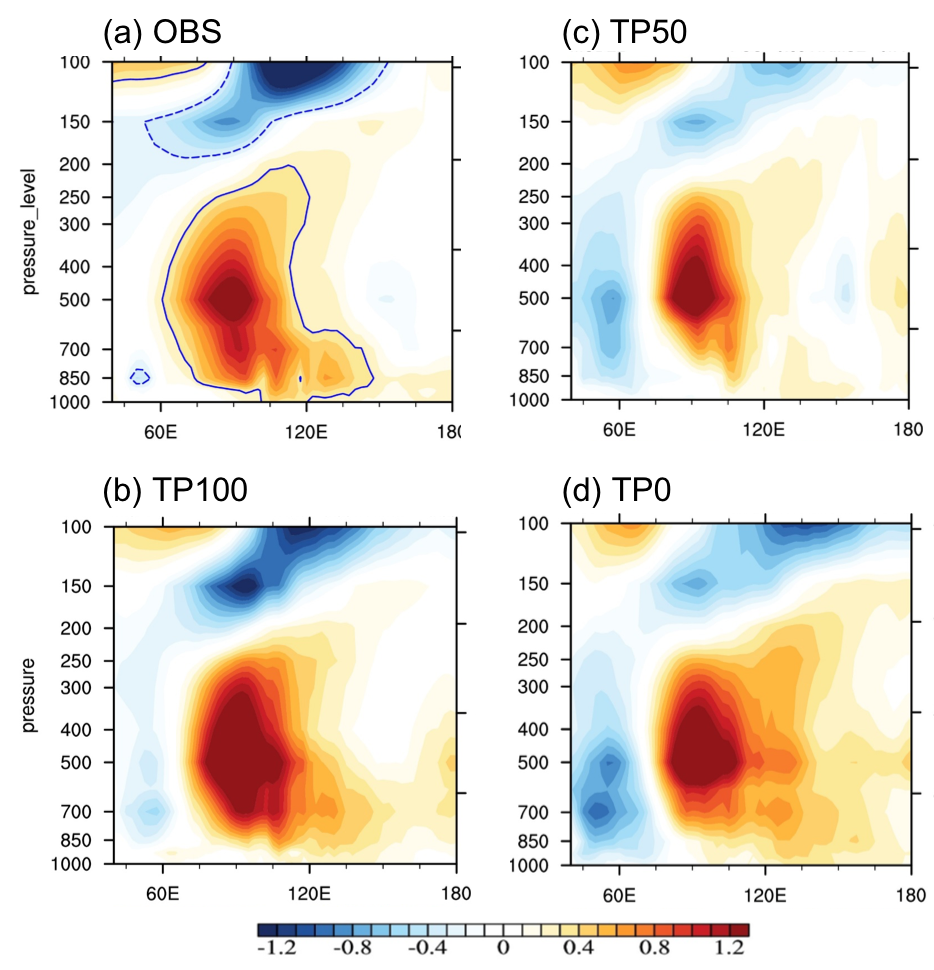


**Figure S5.** Longitude-vertical section of the equatorial zonal asymmetry in equivalent potential temperature (K) averaged between 5°S and 5°N regressed onto the 20-70-day filtered precipitation averaged over the MJO precipitation center (10°S-10°N, 80°-100°E) obtained from (a) observation and model simulations with (b) TP100, (c) TP50, and (d) TP0 during NDJFAM. The regression strengths are scaled to a fixed 3 mm day^-1^ precipitation rate.


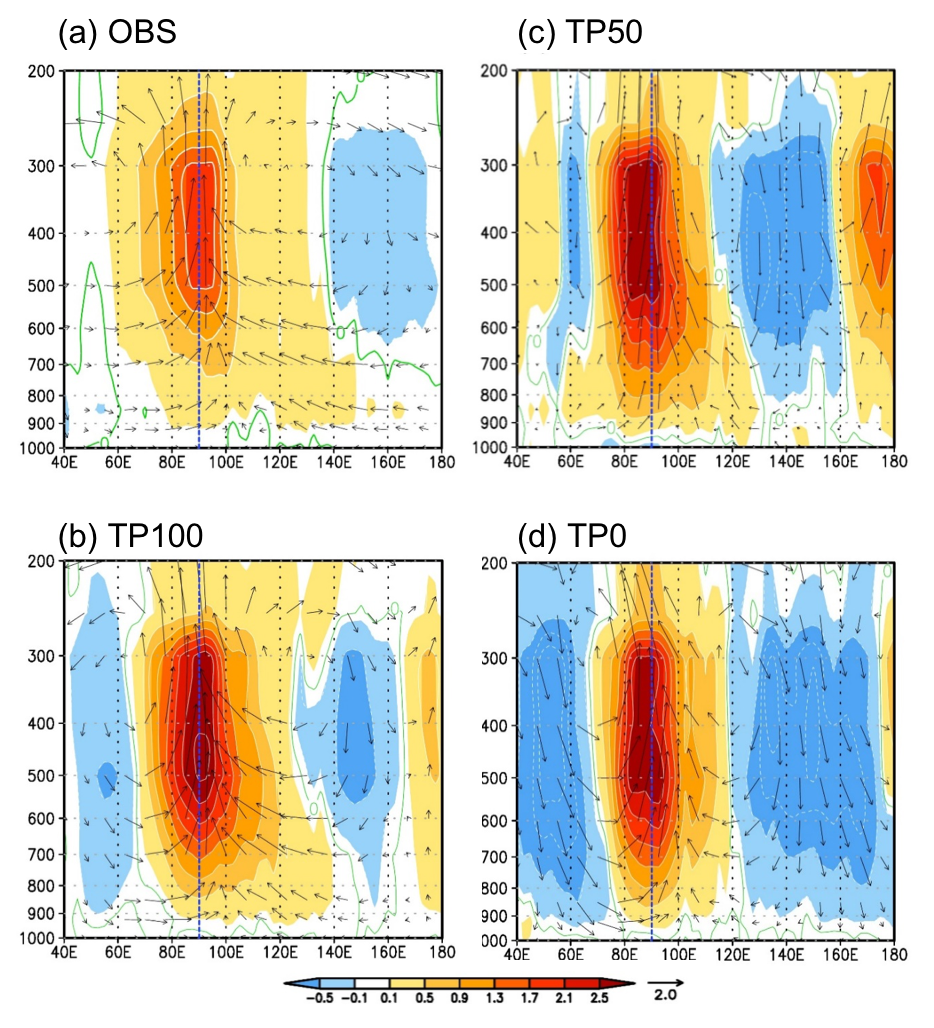


**Figure S6.** Longitude-vertical section of the zonal asymmetry component of the diabatic heating (K day^-1^, shading) and anomalous Walker cell (m s^-1^ for zonal wind and 0.01 Pa s^-1^ for the vertical velocity, vector) along the equator averaged between 5^o^S and 5^o^N obtained from (a) observation and model simulations with (b) TP100, (c) TP50, and (d) TP0. Variables are all regressed onto the 20-70-day filtered precipitation averaged over the MJO precipitation center (10°S-10°N, 80°-100°E). Green contour represents the regression coefficient of 0. The blue vertical lines represent MJO precipitation center (90°E). Only statistically significant areas of diabatic heating are shaded (95% confidence.


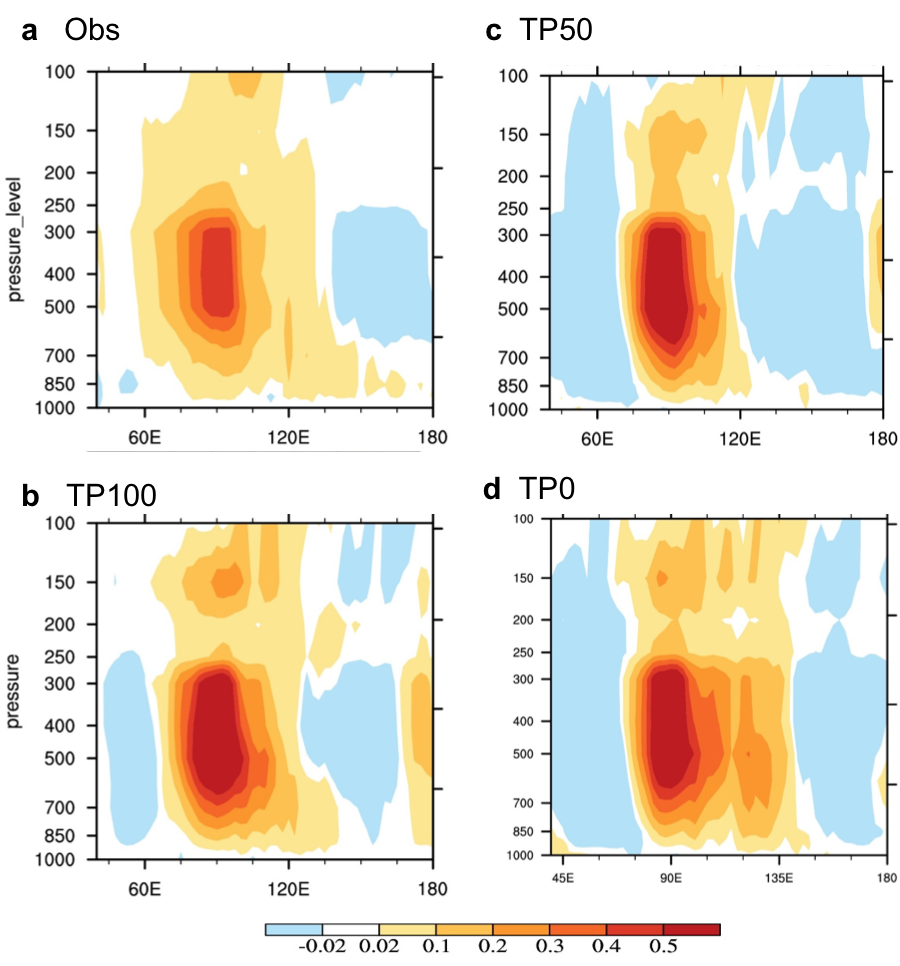


**Figure S7. The MJO available potential energy (APE).** Longitude-vertical section of the MJO APE generation rate (K^2^  day^-1^) along the equator averaged between 5^o^S and 5^o^N regressed onto the 20-70-day filtered precipitation averaged over the MJO precipitation center (10°S-10°N, 80°-100°E) obtained from (a) observation and model simulations with (b) TP100, (c) TP50, and (d) TP0 during NDJFAM. The regression strengths are scaled to a fixed 3 mm day^-1^ precipitation rate.
